# Evaluation of Cytotoxic and Anti-Inflammatory Activities of Extracts and Lectins from *Moringa oleifera* Seeds

**DOI:** 10.1371/journal.pone.0081973

**Published:** 2013-12-09

**Authors:** Larissa Cardoso Corrêa Araújo, Jaciana Santos Aguiar, Thiago Henrique Napoleão, Fernanda Virgínia Barreto Mota, André Luiz Souza Barros, Maiara Celine Moura, Marília Cavalcanti Coriolano, Luana Cassandra Breitenbach Barroso Coelho, Teresinha Gonçalves Silva, Patrícia Maria Guedes Paiva

**Affiliations:** 1 Departamento de Antibióticos, Centro de Ciências Biológicas, Universidade Federal de Pernambuco, Cidade Universitária, Recife, Pernambuco, Brazil; 2 Departamento de Bioquímica, Centro de Ciências Biológicas, Universidade Federal de Pernambuco, Cidade Universitária, Recife, Pernambuco, Brazil; Charité, Campus Benjamin Franklin, Germany

## Abstract

**Background:**

The extract from *Moringa oleifera* seeds is used worldwide, especially in rural areas of developing countries, to treat drinking water. *M. oleifera* seeds contain the lectins cmol and WSMoL, which are carbohydrate-binding proteins that are able to reduce water turbidity because of their coagulant activity. Studies investigating the ability of natural products to damage normal cells are essential for the safe use of these substances. This study evaluated the cytotoxic and anti-inflammatory properties of the aqueous seed extract, the extract used by population to treat water (named diluted seed extract in this work), and the isolated lectins cmol and WSMoL.

**Methodology/Principal Findings:**

The data showed that the aqueous seed extract and cmol were potentially cytotoxic to human peripheral blood mononuclear cells, while WSMoL and diluted seed extract were not cytotoxic. The *M. oleifera* aqueous seed extract and the lectins cmol and WSMoL were weakly/moderately cytotoxic to the NCI-H292, HT-29 and HEp-2 cancer cell lines and were not hemolytic to murine erythrocytes. Evaluation of acute toxicity in mice revealed that the aqueous seed extract (2.000 mg/kg) did not cause systemic toxicity. The aqueous seed extract, cmol and WSMoL (6.25 µg/mL) and diluted seed extract at 50 µg/mL exhibited anti-inflammatory activity on lipopolyssaccharide-stimulated murine macrophages by regulating the production of nitric oxide, TNF-α and IL-1β. The aqueous seed extract reduced leukocyte migration in a mouse model of carrageenan-induced pleurisy; the myeloperoxidase activity and nitric oxide, TNF-α and IL-1β levels were similarly reduced. Histological analysis of the lungs showed that the extract reduced the number of leukocytes.

**Conclusion/Significance:**

This study shows that the extract prepared according to folk use and WSMoL may be non-toxic to mammalian cells; however, the aqueous seed extract and cmol may be cytotoxic to immune cells which may explain the immunosuppressive potential of the extract.

## Introduction

*Moringa oleifera* Lam is one of the fourteen species of the genus *Moringa* (family Moringaceae) found in India, Pakistan, Afghanistan and Bangladesh and is also cultivated in Africa and Latin America [1]. *M. oleifera* seed extract has been used for water treatment, particularly by people in developing countries [2]. 

Studies focusing on the chemical composition of this plant have identified many bioactive substances that may confer diverse pharmacological properties to the preparations obtained from its seeds [3-5]. Although the diluted seed extract is used to treat drinking water, studies investigating additional advantages, such as pharmacological properties, have not previously been published. On the other hand, it has been reported that some substances that are found in the *M. oleifera* seeds are potentially harmful, such as the compounds 4(α-L-rhamnosyloxy) phenylacetonitrile, 4-hydroxyphenylacetonitrile, and 4-hydroxyphenylacetamide, which were isolated from roasted seeds and showed mutagenic effects in a micronucleus assay in mice [6]. 

*M. oleifera* seeds contain bioactive molecules including lectins, proteins of non-immune origin that possess carbohydrate-binding sites able to interact reversibly and specifically with sugars through hydrogen bonding, hydrophobic interactions and Van der Waals forces. These proteins are also known for their ability to agglutinate erythrocytes [7-10]. cMoL (coagulant *M. oleifera* lectin) is a basic protein with 30 kDa and highest activity at pH range 4.0–9.0 while WSMoL (water-soluble *M. oleifera* lectin) is an acidic protein with highest activity at pH 4.5 [11-13]. The lectins from *M. oleifera* seeds show coagulant activity which is responsible for the ability of these seeds to reduce water turbidity [13,14]. cMoL and WSMoL are insecticidal agents that act against *Anagasta kuehniella* and *Aedes aegypti*, respectively [12,15,16] and WSMoL has shown antibacterial activity against species that cause diseases in humans [14]. 

Some lectins are toxic to humans, such as the lectins from *Viscum album* and soybean [17,18]. The toxicity may limit the biotechnological and pharmacological applications of these molecules. WSMoL has been shown not to be genotoxic by the Ames, Kado and cell-free plasmid DNA assays [14,19]. However, there are no other studies about the harmful potential of this lectin to cells of humans.

The pharmacological potential of lectins is broad. Many lectins are active against cancer cell lines and studies reported the antiproliferative activity of them on leukemia and HeLa cell lines [20,21]. Also, it was reported the anti-inflammatory activity of *Caulerpa cupressoides* lectin that reduced leukocyte migration in a murine model of inflammation and *Synadenium carinatum* lectin that reduced the number of inflammatory cells recruited to the lung in a murine model of asthma by acting on key components of the inflammatory response including the transcription factor NF-κB [22,23]. 

This work investigated the cytotoxic effects of the aqueous seed extract, extract used by indigenous populations to treat water for human consumption (named diluted seed extract in this work), and the lectins cMoL and WSMoL from seeds of *M. oleifera* against peripheral blood mononuclear cells and three cancer cell lines (NCI-H292, HT-29 and HEp-2). In addition, this work investigated the hemolytic activity of these same preparations on murine erythrocytes, *in vitro* anti-inflammatory effects (in lipopolyssaccharide (LPS)-stimulated murine macrophages) of the seed extracts and lectins, and the *in vivo* anti-inflammatory activity (in a mouse model of pleurisy) of the aqueous seed extract. Finally, the acute toxicity of the aqueous seed extract was evaluated in mice. These assays were driven by the goal of discovering natural products with promising therapeutic effects and with minimal side effects in humans. 

## Materials and Methods

### Plant material

The seeds of *M. oleifera* were collected in Recife City, State of Pernambuco, northeastern Brazil, and stored at -20 °C. A sample of the collected material is archived, as voucher specimen number 73 345, at the herbarium *Dárdano de Andrade Lima* – IPA (*Instituto Agronômico de Pernambuco*, Recife, Brazil). The authors have authorization from the *Instituto Chico Mendes de Conservação da Biodiversidade* from Brazilian Ministry of the Enviroment for plant collection (number 38690-1). 

### Cell lines and cell culture

The cancer cell lines used for the *in vitro* cytotoxicity tests, NCI-H292 (human pulmonary mucoepidermoid carcinoma), HT-29 (human colon adenocarcinoma) and HEp-2 (human larynx epidermoid carcinoma), were cultivated in DMEM (Dulbecco’s Modified Eagle’s Medium) supplemented with 10% FBS and 100 μg/mL of penicillin–streptomycin-amphotericin B solution at 37 °C in a humidified atmosphere of 95% air and 5% CO2. The cells were obtained from the Cell Bank of Rio de Janeiro, Brazil and maintained in the Laboratory of Cell Culture, Department of Antibiotics at the Federal University of Pernambuco, Brazil. The peripheral blood mononuclear cells (PBMCs) were obtained from peripheral blood from healthy volunteer donors (approved by the Ethics Committee at the Federal University of Pernambuco under the number 0342.0.172.000-11). All participants give written informed consente. Macrophages were collected from the peritoneal cavity of Balb/c mice.

### Animals

Male Balb/c mice weighing 20-25 g were obtained from the Laboratory of Immunopathology *Keizo-Asami* (LIKA) of the Federal University of Pernambuco (UFPE), Brazil. Male Swiss mice (25-30 g) were obtained from the vivarium of the Department of Antibiotics from UFPE. Before use in the experiments, the mice were housed under standardized conditions (22 ± 2 °C, 12-12 h light/dark photoperiod, and 50–60% humidity) and were fed a standard mouse diet with water available *ad libitum*. This study was approved by the Committee for Ethics in Animal Research of the UFPE (process number 23076.029506/2012-64), and the experiments were performed in accordance with the rules of the International Council for Laboratory Animal Science (ICLAS) and the ethical principles of the Brazilian Society of Laboratory Animal Science (SBCAL). The animals subjected to surgery were anesthetized with 0.1 to 0.2 mL/100 g of ketamine and xylazine (2:1). The remaining experiments involving animals were performed using methods designed to minimize pain and suffering.

### Aqueous extract and diluted extract from *M. oleifera* seeds

The aqueous seed extract was prepared according Santos et al. [11] using crushed *M. oleifera* seed and this extract was used to isolate the lectin WSMoL. The resulting powder (10 g) was mixed with distilled water (100 mL) on a magnetic stirrer for 16 h at 4 °C. The mixture was filtered through cotton gauze and centrifuged at 3000 rpm for 15 min. The supernatant was lyophilized using a LIOTOP model L101 freeze dryer (Liobras, São Carlos, Brazil). The diluted seed extract (extract used by indigenous populations to treat water for human consumption) was prepared according to the recommendations of the non-governmental organization ESPLAR (www.esplar.org.br) with the additional inclusion of filtration steps [19]. Macerated shelled seeds (2.0 g) were added to distilled water (100 mL) and were manually agitated for 5 min. The suspension was passed through filter paper. This stock solution (20 μg/μL) was diluted with distilled water to obtain the final concentrations required for each experiment.

### Isolation of cMoL and WSMoL

cMoL was isolated according to the protein isolation procedure described by Santos et al. [13]. *M. oleifera* seed powder (10 g) was extracted with 0.15 M NaCl (100 mL) on a magnetic stirrer for 6 h at 28 °C. After filtration through gauze and centrifugation, the proteins from the saline extract were precipitated by treatment with 60% ammonium sulfate for 4 h at 28 °C. The precipitated protein fraction was dialyzed against distilled water (4 h, 4 °C) followed by 0.15 M NaCl (4 h) and loaded (10 mg of protein) onto a guar gel column (10.0 cm x 1.0 cm) that had previously been equilibrated with 0.15 M NaCl (20 mL/h flow rate). The cMoL was eluted with 1.0 M NaCl and dialyzed against 0.15 M NaCl (4 h, 4 °C). 

WSMoL was isolated according to the protein isolation procedure described by Coelho et al. [12]. *M. oleifera* seed powder (10 g) was extracted with distilled water (100 mL) on a magnetic stirrer for 16 h at 28 °C. After filtration through gauze and centrifugation, the proteins from the extract were precipitated by treatment with 60% ammonium sulfate for 4 h at 28 °C. The precipitated protein fraction was collected by centrifugation, dissolved in 0.15 M NaCl, and dialyzed (3.5 kDa cut-off membrane) against 0.15 M NaCl (6 h at 4 °C). The dialyzed fraction (80 mg of proteins) was then applied to a chitin column (18 x 1.5 cm) that had been equilibrated with 0.15 M NaCl (0.3 mL/min flow rate). After extensive washing with the equilibrating solution, the WSMoL was eluted with 1.0 M acetic acid and was dialyzed against 0.15 M NaCl (6 h at 4 °C).

### Protein content

The protein concentration in all samples was estimated using bovine serum albumin (31-500 μg/mL) as a standard [24]. Briefly, the sample (0.2 mL) was incubated for 10 min at 28 °C with 1 mL of alkaline cupper solution (one part of a 0.5% cupper sulphate solution in 1% sodium citrate plus fifty parts of a 2% sodium carbonate solution in 0.1% sodium hydroxide). Next, 0.1 mL of Folin-Ciocalteus reagent (diluted 1:1 with water) was added and after 30 min the absorbance at 720 nm was recorded.

In the chromatography steps, protein elution was monitored by measuring the absorbance at 280 nm.

### Hemagglutinating activity

The assay was conducted in microtiter plates (Kartell S.P.A., Italy) using a suspension (2.5% v/v) of rabbit erythrocytes treated with glutaraldehyde [25]. Hemagglutinating activity was determined by mixing a twofold serial dilution of sample (50 μL) with 0.15 M NaCl in microtiter plates. Next, the erythrocyte suspension (50 μL) was added to each well, and the assay was incubated for 45 min at 27 °C. One hemagglutination unit was defined as the reciprocal of the highest dilution of the sample able to promote full erythrocyte agglutination. Specific hemagglutinating activity was defined as the ratio of the hemagglutinating activity to the protein concentration (mg/mL) [26]. The assay was also performed at presence of 0.2 M fructose or 0.5 mg/mL thyroglobulin (inhibitors of WSMoL hemagglutinating activity) by incubating (15 min, 28 °C) the extracts with inhibitors before addition of erythrocytes. 

### Evaluation of seed extracts for saponin

Saponin detection was performed by a qualitative test [27]. The aqueous seed and the diluted seed extracts were vigorously agitated for two minutes and allowed to stand for two hours. The persistence of foam at the end of the rest period was considered a positive result. We also performed a capillary flow test to compare the flow rate of the extracts with that of distilled water. The extracts with a flow rate greater than that of water were considered to contain saponins.

### Evaluation of the cytotoxicity of the seed extracts and lectins on peripheral blood mononuclear cells (PBMCs)

PBMCs were purified from human blood by gradient separation using the Histopaque-1077 solution for cell separation (Sigma Aldrich, St. Louis, Missouri, EUA). The cells were isolated from a 6 mL blood sample in 5 mL of phosphate buffered saline (PBS) to which 3 mL of Ficoll Histopaque-1077 was added. After centrifugation at 1500 rpm for 30 min, the mononuclear cells (present in the intermediate region between the red cells and serum) were aspirated. The lymphocyte suspension was transferred to another tube to which PBS was added to a total volume of 11 mL, and the resulting suspension was centrifuged for 20 min at 1000 rpm. The supernatant was then discarded, and the lymphocyte pellet was resuspended to a final concentration of 3 x 10^5^ cells/mL in 2 mL of RPMI 1640 medium supplemented with 20% fetal bovine serum, 100 IU/mL penicillin and 100 μg/mL streptomycin. Lymphocyte proliferation was induced by the addition of 3% phytohemagglutinin, a mitogen that acts on T lymphocytes, which are the predominant cell population in this assay [28]. 

To determine the cytotoxicity with PBMCs, the *M. oleifera* samples were added to the cell cultures in graded concentrations (aqueous seed extract: 0.78–50 μg/mL; diluted seed extract: 6.25–400 μg/mL; cMoL and WSMoL: 1.56–100 μg/mL) and were incubated for 72 h at 37 °C in a humidified atmosphere of 95% air and 5% CO2. Next, MTT was added (25 μL, 0.5 mg/mL), the microplates were placed in an incubator for 3 h, and then DMSO was added (100 μL to each well). The optical density of the wells was measured at 540 nm. The concentration that inhibited 50% of the cell growth compared with the control (IC50) was determined. Two independent experiments were performed in duplicate. The samples with an IC50 < 10 μg/mL were considered to be very toxic, whose with an IC50 between 10 and 100 μg/mL were considered to be potentially toxic, and those with an IC50 > 100 μg/mL were considered to be non-cytotoxic [29,30].

### Evaluation of the cytotoxicity of the aqueous seed extract, cMoL and WSMoL on cancer cell lines

Cell viability was measured using an MTT reduction assay, which is based on the conversion of 3-(4,5-dimethyl-2-thiazole)-2,5-diphenyl-2H-tetrazolium bromide (MTT) to a formazan product by the action of the enzyme succinyl dehydrogenase, which is present in the mitochondria of viable cells [31,32]. The NCI-H292, HT-29 and HEp-2 cells (10^5^ cells/mL) were plated in DMEM medium in 96-well microplates and incubated for 24 h at 37 °C. After this period, 25 μL of the aqueous seed extract (50 μg/mL) or cMoL or WSMoL (25 μg/mL) were added to each well and incubated for 72 h. Next, MTT dye (25 μL; 0.5 mg/mL) was added to the wells, and the assay was incubated for another 3 h. After this period, the medium was removed and dimethylsulfoxide (DMSO, 100 μL) was added to the wells to solubilize the formazan salts. The optical density of the wells was measured at 540 nm, and was compared to those in the control wells (cells incubated only with medium). Two independent experiments were performed in duplicate. The samples were classified by the percent decrease in cell viability and classified as follows: inactive (1-20%), weakly active (20-50%), moderately active (50-70%) or very active (70-100%) [33].

### Hemolytic Assay

The hemolytic assay was performed in 96-well microplates. A 0.85% NaCl solution containing 10 mM CaCl2 was added to each well. Samples (50 μL) of aqueous seed extract or diluted seed extract at 15.62 to 2000 μg/mL in 5% DMSO or cMoL or WSMoL at 0.19 to 25 μg/mL in 0.15 M NaCl were added to the wells containing 100 μL of the saline solution in a 1:2 dilution. Each well received 100 μL of a 2% (v/v) suspension of mouse erythrocytes in 0.85% saline containing 10 mM CaCl2. In the negative controls, 100 μL of the saline solution plus 50 μL of the saline solution or 50 μL of 5% DMSO were plated. The positive control (to obtain 100% hemolysis) contained 80 μL of saline solution plus 20 μL of 0.1% Triton X-100 in 0.85% saline. After centrifugation for 1 h followed by incubation for 1 h at 27 °C, the supernatant was discarded, and the released hemoglobin was measured by absorbance at 450 nm. Two independent experiments were performed in duplicate. The effective concentrations that resulted in 50% hemolysis (EC50) when compared with that observed in the positive controls were only considered to be active extracts when the EC50 < 200 μg/mL [34].

### Evaluation of the acute toxicity of the aqueous seed extract

The acute toxicity of the aqueous seed extract was determined according to the guidelines of the Organization for Economic Cooperation and Development (OECD) for the testing of chemicals, n° 423, adopted on December 17, 2001. Female Swiss albino mice (n=3 for each group) received a single dose of the aqueous extract (2000 mg/kg) or saline solution, as a control. The animals were observed individually during the first hour and at 2, 12 and 24 h after administration to determine the time of death or to investigate possible toxic effects. For the following 14 days the animals were observed once daily. A range of signs such as general activity, irritability, touch response, contortions, tremors, convulsions, tachycardia, piloerection, stereotyped movements, somnolence, defecation, diarrhea and miction were analyzed. The daily consumption of water and food as well as the body weight of the mice during the experiment were also recorded. On the 14th day after administration of the extract, the animals were anesthetized with 0.1 to 0.2 mL/100 g of ketamine and xylazine (2:1), and blood was collected by cardiac puncture and placed in tubes with anticoagulant (EDTA). Hematological index were determined using an automated Horiba ABX Micros 60-Horiba analyzer. The organs (liver, kidney and spleen) were analyzed macroscopically and dried and weighed to calculate the index of organs, which is given by the following formula: *Organ weight* (*mg*)*/ Body Weight* (*g*).

### Effect of the *M. oleifera* extracts and lectins on the viability of LPS-stimulated peritoneal macrophages

Balb/c mice received 2.5 mL of 3% sodium thioglycollate by intraperitoneal injection. After 72 hours, the animals were euthanized in a CO2 chamber and the peritoneal exudate was collected by washing the cavity with cold sterile PBS (5 mL). The viability of the adherent cells was assessed using a trypan blue exclusion test, and the cell suspension (5x10^6^ cells/mL) was cultured in microplates containing RPMI-1640 medium supplemented with 10% FBS, antibiotic solution (1000 UI/mL penicillin and 100 mg/L streptomycin), and 200 mM L-glutamine for 2 h at 37 °C in a 5% CO2 atmosphere. Subsequently, the plates were washed to remove all non-adherent cells. Fresh media containing 100 μL of the aqueous seed extract (6.25–50 μg/mL), the diluted seed extract (50–400 μg/mL) or cMoL or WSMoL (6.25–100 μg/mL) in the presence of LPS (1 μg/mL) was added to the macrophages adhered onto the plate. After incubation at 37 °C in an atmosphere of 5% CO2 for 24 h, the supernatant was collected for further analysis of the nitrite concentration and cytokine production, and the viability of the cells was assessed using an MTT assay. Each concentration was tested in quadruplicate. Three independent experiments were performed, and only concentrations at which the percentage of viable cells was greater than 70% were used to assess the in vitro anti-inflammatory activity [35].

### Evaluation of the *in vivo* anti-inflammatory effects of the aqueous seed extract on carrageenan-induced pleurisy

Animals were pretreated orally with the aqueous seed extract (125, 250 or 500 mg/kg), dexamethasone (0.5 mg/kg) or the saline solution (NaCl 0.85%) in groups of six randomly assigned animals. One hour after treatment, the animals were anesthetized with 0.1 to 0.2 mL/100 g of a combination of 50% ketamine and 2% xylazine (2:1). Pleurisy was induced by the surgical administration of 0.1 mL of the phlogistic agent carrageenan (1%) into the pleural cavity. Four hours after the induction of inflammation, the animals were euthanized, the thorax was opened, and the pleural cavity was washed with 1.0 mL of sterile PBS containing heparin (20 IU/ mL). Samples of the pleural lavage fluid were collected for evaluation of the cytokine and NO levels as well as cell migration. Fragments of the lung were also collected to evaluate myeloperoxidase activity, and for histopathological analysis [36]. The cell counts were performed on an automated ABX micros 60-Horiba analyzer. At the end of pleurisy the lung fragments were collected and fixed in 10% buffered formalin. The tissue was dehydrated with graded ethanol, embedded in paraffin blocks and sectioned. The sections were stained with hematoxylin-eosin for examination under light microscopy.

### The analysis of nitrite and the measurement of cytokines

The nitrite present in the supernatant of the macrophage cultures and in the pleural exudate was used as an indicator of NO production using the Griess reaction. Briefly, the samples (50 μL) were mixed with an equal volume of Griess reagent in a 96-well microtiter plate and were incubated at room temperature for 10 min. The absorbance was read at 540 nm using a microplate reader and the nitrite concentrations were determined by comparison with a standard curve of sodium nitrite. The results were expressed in μM. 

The concentrations of TNF-α, IL-1β and IL-6 were measured using sandwich ELISA kits specific for mice (eBioscience, San Diego, California, USA) according to the manufacturer’s instructions. The results were expressed as pg/mL.

### Myeloperoxidase activity assay

A myeloperoxidase activity assay was used to evaluate polymorphonuclear leukocyte accumulation. Lung tissues were collected and weighed 4 h after the carrageenan injection. Each fragment of the organ was homogenized in a solution containing 0.5% (w/v) hexadecyltrimethylammonium bromide (HTAB) that had been dissolved in 10 mM potassium phosphate buffer (pH 7.0). The homogenate was centrifuged for 5 min at 7000 rpm, and an aliquot of the supernatant was allowed to react with a solution of hydrogen peroxide and o-dianisidine in potassium phosphate buffer. The myeloperoxidase activity was measured by the rate of change in absorbance at 450 nm after 5 min of incubation [36].

### Statistical Analysis

For the cell viability analysis, the IC50 was calculated from nonlinear regression using GraphPad Prism v. 5.0 software. For the remaining tests, one-way ANOVA followed by the Newman-Keuls test was used to evaluate the differences among the treatments. P values < 0.05 were considered to be statistically significant.

## Results and Discussion

### Protein, Hemagglutinating activity and Saponin evaluation

Characterization of the *M. oleifera* extracts reveals that the aqueous seed extract contains 1.52 mg/mL of protein and shows a specific hemagglutinating activity of 42.1, while the diluted seed extract contains 0.0486 mg/mL of protein and shows a specific hemagglutinating activity of 2634. Thus, although the diluted seed extract has a low concentration of protein, its high hemagglutinating activity indicates a high concentration of lectins. The hemagglutinating activity of extracts was inhibited by fructose and neutralized by thyroglobulin indicating that WSMoL was the component responsible for hemagglutination. 

Studies have reported presence or absence of saponins in extracts from *M. oleifera* seeds [37,38] and thus, aqueous seed and the diluted seed extracts were investigated for this class of compound which usually shows hemolytic activity. Saponins were not detected in the extracts since foam was not observed in the test tubes and the flow rates of extracts were the same of water in the capillary flow test. 

### Evaluation of the cytotoxicity of the *M. oleifera* extracts and the lectins on peripheral blood mononuclear cells (PBMCs)

The maintenance of the integrity of PBMCs is essential for the body's defense against attack by pathogens and in an inflammatory response [39,40]. *M. oleifera* seeds contain isothiocyanates [41] that are toxic compounds to normal cells. In the present work, PBMCs were used to evaluate the cytotoxicity of the extracts and the lectins from *M. oleifera* seeds. PBMCs are frequently used to evaluate the cytotoxicity to normal cells of natural or synthetic products [42]. The aqueous seed extract and cMoL were potentially cytotoxic for PBMCs (Table 1), with IC50 measurements of 34.3 ± 2.31 μg/mL and 11.72 μg/mL, respectively. On the other hand, treatment for 72 h with WSMoL at the tested concentrations did not alter cell viability. The IC50 of 144 μg/mL determined for the diluted seed extract, which is used to treat drinking water, indicate that it was not cytotoxic to PBMCs. The results showed that the diluted seed extract is safe while the aqueous seed extract, a preparation with higher protein concentration, may potentially be harmful indicating that the population should not indiscriminately increase the concentration of the extract to treat water. 

**Table 1:** Cytotoxicity on human peripheral blood mononuclear cells and hemolytic activity of *M. oleifera* seed extracts and lectins.

	Cytotoxicity on human peripheral blood mononuclear cells	Hemolytic activity
**Treatment**	**IC_50_ (µg/mL)**	**EC_50_ (µg/mL)**
Aqueous seed extract	34.3 ± 2.31	>2000
Diluted seed extract	144.8 ± 1.56	>2000
cMoL	11.72 ± 1.51	> 25
WSMoL	>100	> 25

This result is likely due to the low concentration of toxic compounds in the diluted seed extract; therefore, the preparation of extracts using an amount of seeds higher than that used for water treatment is not recommended. Rolim et al. [19] also showed that the *M. oleifera* seed extract was mutagenic in the Ames and Kado assays at concentrations 3, 4, 5 and 7.5- fold higher than that used by population to treat water. 

In this study, none of the tested preparations was effectively cytotoxic to lymphocytes suggesting that the extracts and lectins from *M. oleifera* seeds may be used in a wide range of pharmacological studies. This result represents an additional stimulus to the use of the extract by population. Since the extracts and lectins were not harmful to human cells, we investigated their pharmacological potential by determining the toxicity to cancer cells as well as their anti-inflammatory activity.

### Evaluation of the cytotoxicity of the aqueous seed extract and lectins on cancer cell lines

In this work the cytotoxicity on the cancer cell lines was performed by treatment with a single concentration of the aqueous seed extract (50 μg/mL), cMoL (25 μg/mL) and WSMoL (25 μg/mL). The diluted seed extract was not evaluated because it was not cytotoxic to PBMCs and a more concentrated extract (aqueous seed extract) had already been included among the samples tested. The aqueous seed extract inhibited the growth of NCI-H292 (42.7%), Hep-2 (24.9%) and HT-29 (51.3%) cells, but the lectins showed no activity against growth of the HT-29 cells. cMoL inhibited NCI-H292 growth (33.9%) to a greater degree than it did Hep-2 (25.0%) growth, while WSMoL had a similar inhibitory effect on NCI-H292 (38.7%) and Hep-2 (32.6%) cell growth. Other concentrations were not evaluated due to the low anticancer activity detected. The percent decrease in cell viability revealed that aqueous seed extract and the lectins cMoL and WSMoL were weakly active on NCI-H292 and Hep-2 while the aqueous seed extract was moderately active and the lectins were weakly active on HT-29. The low cytotoxic activity of aqueous extract, cMoL and WSMoL detected here does not exclude their potential uses as cytotoxic agent on other cancer cell lines because genetic differences can lead to the expression of distinct membrane receptors that can promote the activation of altered signal transduction cascades in other similarly treated cell types [43]. 

### Hemolytic Assay

In the search for new substances that have promising pharmacological activity, and do not cause harmful effects to the body, trials investigating the ability of synthetic or natural products to cause damage to erythrocyte membranes are frequently used [44,45]. The hemolytic assay was performed to assess the potential of the *M. oleifera* seed extracts and lectins to cause injury to the plasma membrane of the cells, either by forming pores or by causing their total collapse. 

Table 1 show that both the aqueous seed and the diluted seed extracts did not cause hemolysis even at concentrations of up to ten times greater than those used by indigenous populations to treat water for human consumption. Similarly, neither cMoL nor WSMoL induced hemolysis at any of the concentrations tested. Although the aqueous seed extract and cMoL were potentially cytotoxic to PBMCs, none of the preparations evaluated in this work showed hemolytic activity and thus do not damage the plasma membrane of erythrocytes. 

### Acute toxicity

Before the evaluation of *in vivo* anti-inflammatory activity of the aqueous seed extract, it was evaluated for acute toxicity in Swiss albino mice to determine the proper dose for the *in vivo* anti-inflammatory assay. In the first hours and for the following 14 days after administration of the extract (2000 mg/kg), no signs of systemic toxicity were observed, and all the animals survived. Table 2 shows that there were no significant differences (p<0.05) in body weight between the control and treated groups. Additionally, there was no change in the consumption of food and water, and the organs indices were not different between the groups. However, there was a reduction in the number of erythrocytes, leukocytes, platelets, hemoglobin and hematocrit (Table 3).

**Table 2:** Effect of *M. oleifera* seed extracts and lectins on the viability of LPS-stimulated peritoneal macrophages.

		**Viable cells (%)**		
**Concentration (µg/mL)**	**Aqueous seed extract**	**Diluted seed extract**	**cMoL**	**WSMoL**
**6.25**	77.56+4.3	NT	89.47+2.3	71.5+2.15
**12.5**	64.98+1.3	NT	66.4+0.9	73.8+1.7
**25**	57.95+0.5	NT	57.38+0.6	56.0+0.1
**50**	56.95+0.2	85.33+1.0	32.2+1.4	53.6+1.8
**100**	NT	57.9+1.6	14.4+0.8	17.5+1.5
**200**	NT	46.7+2.0	NT	NT
**400**	NT	23.3+0.9	NT	NT

Data are presented as mean ± standard deviation of three independent experiments. NT: not tested.

**Table 3:** Effect of *M. oleifera* seed extracts and lectins (cMoL and WSMoL) on TNF-α, IL-1β and IL-6 production by murine macrophages stimulated with LPS.

**Treatment (µg/mL)**	**TNF-α (pg/mL)**	**IL-1β (pg/mL)**	**IL-6 (pg/mL)**
Control	52.3±2.2	34.6±2.6	351.4±33.7
LPS ( 1 µg/mL)	732.5+13.7^*^	577.5±3.3^*^	410.4±23.8^*^
Aqueous seed extract (6.25 µg/mL)	100.0+19.3^*#^	186.28±48.4^*#^	401.6±8.0^*^
Diluted seed extract (50 µg/mL)	305.8±7.4^*#^	266.29±39.4^*#^	419.3±0.3^*^
cMoL (6.25 µg/mL)	351.1+1.8^*#^	114.72±7.3^*#^	403.8±18.1^*^
WSMoL (6.25µg/mL)	492.2+65.2^*#^	726.67±41.5^*#^	402.0±2.7^*^

Data are presented as mean ± standard deviation. *p<0.05 compared to control (cells in medium culture only) by ANOVA followed by Newman-Keuls test. #p<0.05 compared to LPS by ANOVA followed by Newman-Keuls test.

We also investigated the acute toxicity of the aqueous seed extract at a dose of 500 mg/kg and there were no statistically significant differences in any measured outcomes when treated mice were compared with controls. 

Despite the slight decreases in the numbers of erythrocytes, platelets, hemoglobin and the hematocrit that were detected in the acute toxicity assay of the aqueous seed extract treatment, these values all remained within normal range [46] indicating that the aqueous seed extract did not cause toxicity at the systemic level. Mahajan and Mehta [47] detected immunosuppressive activity in ethanolic *M. oleifera* seed extract. Our data revealed that immunosuppressive components were not solubilized by water or were present in concentrations insufficient to promote damage. However, alterations in the number of erythrocytes, leukocytes, platelets, hemoglobin and hematocrit indicate that it is need care with increasing concentration of the extract used by population. 

In this work, the doses selected for the *in vivo* anti-inflammatory assay were 125, 250 and 500 mg/kg for the aqueous seed extract, which were 16-, 8- and 4-fold lower, respectively, than those used in the acute toxicity assay. An LD50 of 446.5 mg/kg in mice (i.p. route) has been reported for the extract prepared by mixing the powder of one seed (approximately 0.2 g) in 10 mL of distilled water after stirring for 60 minutes [48]. In our study there were no observed deaths even though we used a higher dose of extract (2000 mg/kg). Different routes of administration were used, and it is likely that the digestion and metabolism that occurs when the extract is delivered orally may have reduced the toxicity of the aqueous seed extract.

### *In vitro* anti-inflammatory activity of the *M. oleifera* seed extracts and lectins

 Bacterial endotoxins, such as LPS, activate macrophages, leading to the production of several molecules involved in the inflammatory process. Among these is NO [49], which is an important proinflammatory mediator associated with the activation of T lymphocytes and the increased vascular permeability observed in inflammatory processes [50,51]. The *M. oleifera* seed extracts and lectins were evaluated for their *in vitro* anti inflammatory activity using LPS-stimulated murine peritoneal macrophages. 

First, the cytotoxicity of the samples was evaluated (Table 4) to avoid the use of cytotoxic concentrations for the analysis of their anti-inflammatory effects and, consequently, false positive results. The aqueous and diluted seed extracts, cMoL and WSMoL all affected cell viability in a dose-dependent manner. Treatment with cMoL promoted weak cytotoxic effects at 6.25 μg/mL. However, WSMoL has not been cytotoxic at the 6.25 and 12.5 μg/mL concentrations; thus, the lowest concentration was selected for the next assay. The aqueous seed extract at a concentration of 6.25 μg/mL and the diluted seed extract at 50 μg/mL had the smallest effect on cell viability. 

**Table 4:** Effect of *M. oleifera* aqueous seed extract (2000 mg/kg) on physiological parameters of mice 14 days after administration.

			**Animal weight (g)**		**Index of organs (mg/g)**		
**Group**	**Feed intake (g)**	**Water consumption (mL)**	**Initial**	**Final**	**Liver**	**Kidney**	**Spleen**
**Extract**	16.85±2.88	28.46±4.73	29.56±0.64	32.86±1.47	61.60±1.65	4.85±0.12	5.78±0.50
**Control**	16.38±2.51	28.12±3.72	29.4±1.6	31.2±2.3	60.02±2.45	5.41±0.27	6.76±0.42

Data are presented as mean ± standard deviation, with 3 animals per group. No statistical differences (p>0.05) were identified by ANOVA followed by Newman-Keuls test.

An infusion (10 g of seed powder in 100 mL of distilled water) of *M. oleifera* seeds has previously been shown to reduce edema formation in rats, supporting their anti-inflammatory activity [52]. Similarly, β-sitosterol, a compound with potent activity against airway inflammation, whose mechanism of action includes reducing the production of TNF-α, was isolated from *M. oleifera* seeds [53]. Thus, we evaluated the *in vitro* anti-inflammatory activity of the aqueous seed and the diluted seed extracts and cMol and WSMoL using LPS-stimulated murine macrophages by measuring the levels of the mediators involved in the inflammatory process. 

**Figure 1:**
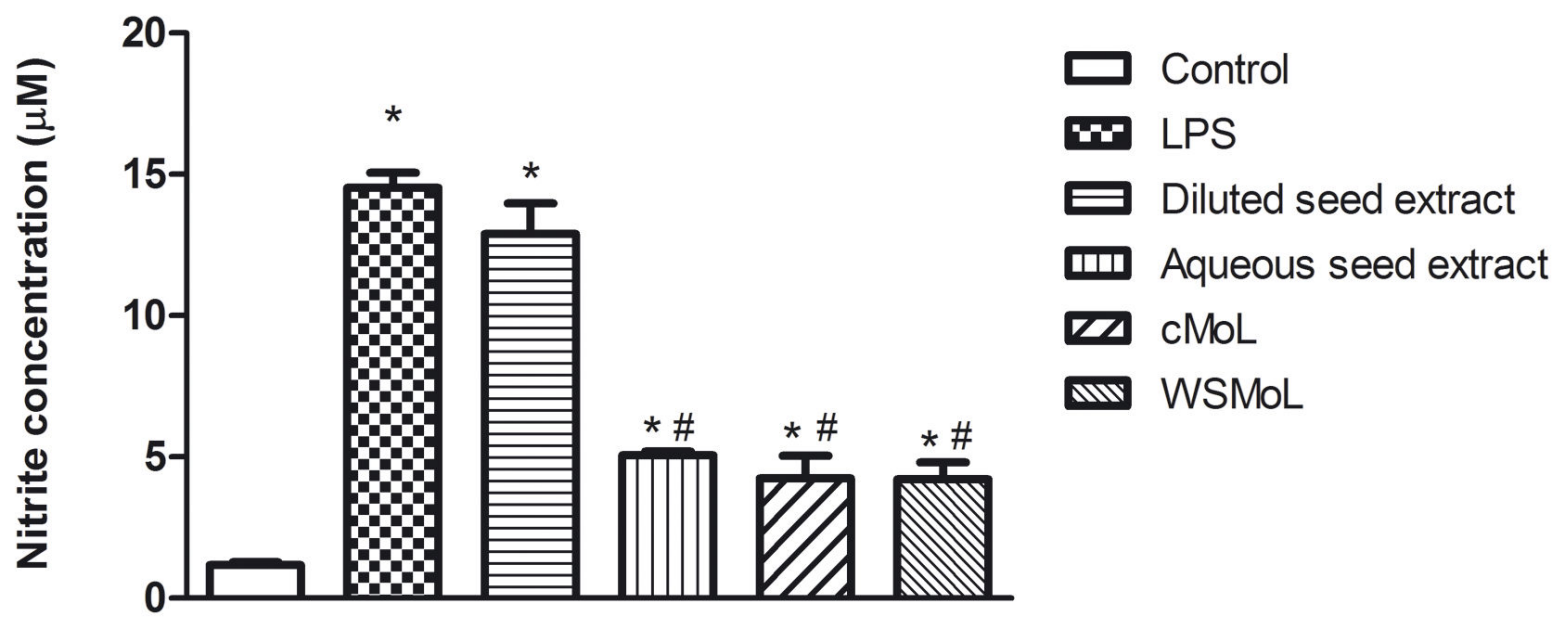
Effect of *M. oleifera* seed extracts and lectins (cMoL and WSMoL) on nitric oxide production by murine macrophages stimulated with LPS. Data are presented as mean ± standard deviation of three independent experiments. *p<0.05 compared to control (cells in medium culture only) by ANOVA followed by Newman-Keuls test. #p<0.05 compared to LPS by ANOVA followed by Newman-Keuls test.

The aqueous seed extract (6.25 μg/mL) was able to reduce NO production, while the diluted seed extract (50 μg/mL) did not cause a statistically significant reduction of this mediator (Figure 1). Both lectins (6.25 μg/mL) were able to reduce NO production by macrophages stimulated with LPS when compared with cells that had been exposed only to lipopolysaccharide. These results indicate that the *in vitro* anti-inflammatory activity of the aqueous seed extract and both lectins is due, at least in part, to the regulation of NO production. 

Once TNF-α plays a crucial role on inflammatory process due to its ability to stimulate the production of other proinflammatory cytokines [54] it was tested if the extracts and lectins may reduce the production of this cytokine. In addition we determined the levels of IL-1β since the TNF-α stimulates release of this cytokine which in turn stimulates the activation of NFκB promoting increase on production of other proinflammatory cytokines [55,56].

Table 5 shows the data from the measurements of cytokine production. The aqueous seed, diluted seed extracts and cMoL significantly reduced (p<0.05) the levels of TNF-α and IL-1β that were released by LPS-stimulated macrophages. WSMoL reduced the production of TNF-α and increased the levels of IL-1β. None of the tested *M. oleifera* samples affected the IL-6 levels. 

**Table 5:** Effect of aqueous seed extract (2000 mg/kg) on hematological parameters of mice 14 days after administration.

**Parameter**	**Extract**	**Control**
Red blood cells (10^6^/mm^3^)	8.10±0.96*	11.17±0.30
Leukocytes (10^3^/mm^3^)	8.06±0.62*	12.5±1.0
Platelets (10^3^/mm^3^)	72.66±12.6*	250±9.19
Hemoglobin (g/dL)	14.66±0.15*	17.8±1.27
Hematocrit (%)	42.76±3.97*	57.2±4.8
VCM (μμg)	52.33±0.57	51±2.82
HCM (pg)	16.73±0.25	15.75±0.49
CHCM (%)	31.33±1.36	31.1±0.28

Data are presented as mean ± standard deviation, with 3 animals per group. *p<0.05 with compared to control by ANOVA followed by Newman-Keuls test.

LPS promotes the activation of NF-κB in macrophages and stimulates the production of NO and pro-inflammatory cytokines. Therefore, the reduction of NO levels may be related to the inhibition of activation of nuclear transcription factor κB, which regulates the expression of iNOS and of genes related to the production of cytokines [57]. This is supported by the observed inhibition of TNF-α and IL-1β by the aqueous seed extract and cMoL, both *in vitro* and *in vivo*. 

The reduction of NO levels induced by the aqueous seed extract could be due to the synergistic effect of its constituents, including the lectins. However, the diluted seed extract did not reduce the levels of NO, although it significantly reduced the production of TNF-α and IL-1β. The mechanism of action of this extract may be due to the inhibition of NF-κB activation, and the absence of activity with respect to NO production suggests the translational or post-translational regulation of iNOS. However, further studies should be conducted to confirm this hypothesis. 

TNF-α is largely responsible for LPS-induced nitric oxide production, as shown by Steege et al. [58], who demonstrated this using a combination of anti-TNF-α and anti-IFN-γ antibodies. Thus, the reduction in NO levels observed in this study may be due to the marked reduction in the levels of TNF-α. 

The activation of macrophages with LPS can also be associated with cytokine production via p38 MAPK, mainly by MAP kinase kinase 3 (MKK3) and MKK6. It has previously been demonstrated that MKK3 regulates IL-1β but not TNF-α [59]. Despite having reduced the production of TNF-α, WSMoL failed to reduce the levels of IL-1β. Therefore, this suggests that WSMoL is activating MKK3. Similarly, the extracts and cMoL may also be regulating the p38 MAPK pathway. 

It has previously been demonstrated that IL-6 may control the production of proinflammatory cytokines, thereby maintaining their synthesis below harmful levels [60]. The failure of the aqueous seed, diluted seed extracts as well as cMoL and WSMoL to reduce IL-6 levels may explain why there was no increase in the levels of TNF-α and IL-1β.

### Evaluation of the *in vivo* anti-inflammatory effects of the aqueous seed extract on carrageenan-induced pleurisy

Once that the more concentrated extract (aqueous seed extract) did not cause acute toxicity, we choose it for evaluation of anti-inflammatory activity *in vivo*. Carrageenan is a widely used phlogistic agent that promotes inflammation and is associated with increased NO levels, leukocyte migration and fluid extravasation [61]. 

Models of carrageenan-induced pleurisy have been widely employed to investigate the pathophysiology of acute inflammation and to evaluate the efficacy of drugs in inflammation [62]. This model can be divided into two phases, the first (4 hours after induction of inflammation) is characterized by the predominance of neutrophils, while the second (48 hours after induction of inflammation) shows predominantly mononuclear cells in the pleural cavity [63]. Furthermore, this pleurisy mimics the inflammatory process that occurs in asthmatic patients [64], and neutrophils are among the cells found in abundantly in asthma [65].

**Figure 2:**
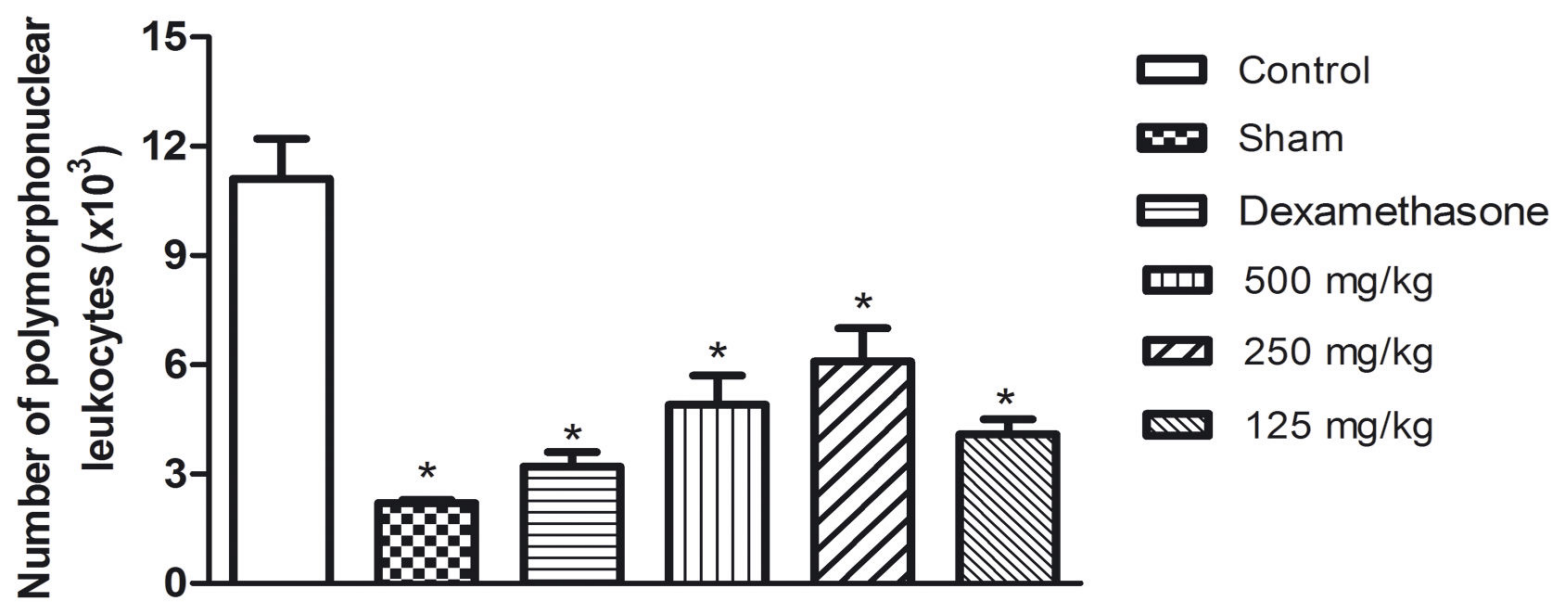
Number of polymorphonuclear leukocytes (x10^3^) in exudate of the pleural cavity after 4 h of pleurisy induction by carrageenan in mice previously treated with aqueous seed extract. Data are presented as mean ± standard deviation of groups with 6 animals. *p<0.05 in comparison with control by ANOVA followed by Newman-Keuls test.

The oral administration of the aqueous seed extract at the selected doses before the induction of pleurisy by carrageenan caused a reduction in leukocyte migration in a dose-dependent manner, as shown in Figure 2. All of the doses tested showed effectiveness in reducing cell migration, and the highest percentage of inhibition (71.2%) was observed at a dose of 500 mg/kg.

**Figure 3:**
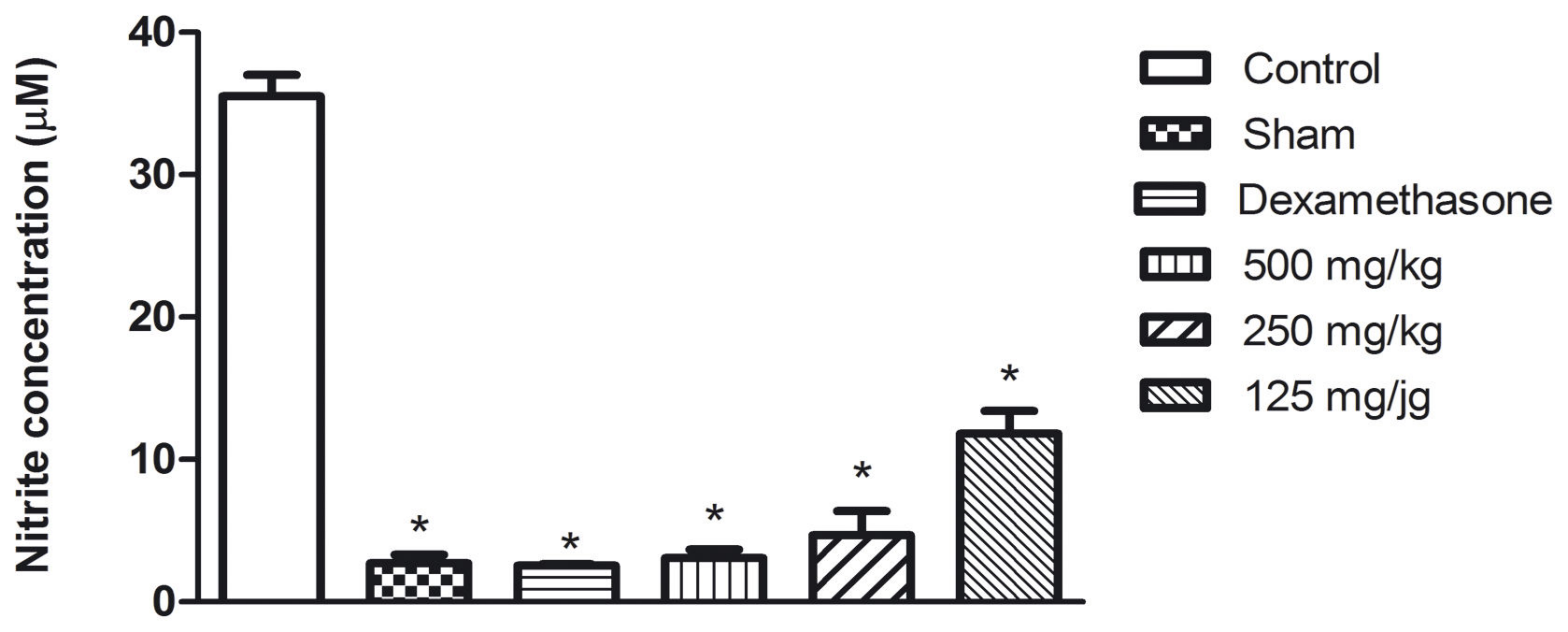
Effect of aqueous seed extract on nitric oxide production (µM) in the assay of carrageenan-induced pleurisy. Data are presented as mean ± standard deviation of groups with 6 animals. *p<0.05 in comparison with control by ANOVA followed by Newman-Keuls test.

The aqueous seed extract reduced the production of NO with percentages of inhibition of up to slightly more than 90% compared with the controls (Figure 3). This result corroborates that obtained in the *in vitro* anti-inflammatory assay and demonstrates that the reduction of NO production may be involved in the mode of action of the extract against acute inflammation induced by carrageenan. 

**Figure 4:**
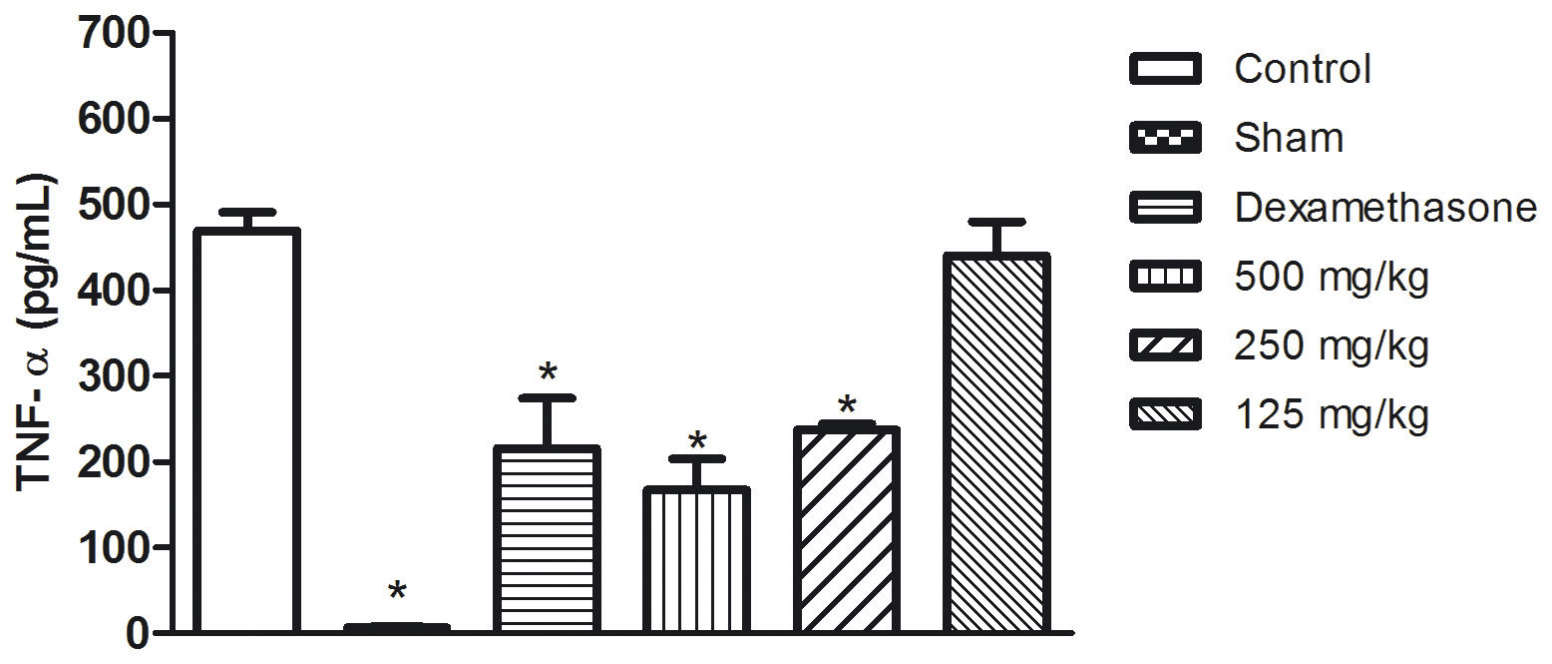
TNF-α (pg/mL) levels after the end of carrageenan-induced pleurisy assay in exudate of the pleural cavity of mice previously treated with aqueous seed extract. *p<0.05 in comparison with control by ANOVA followed by Newman-Keuls test.

**Figure 5:**
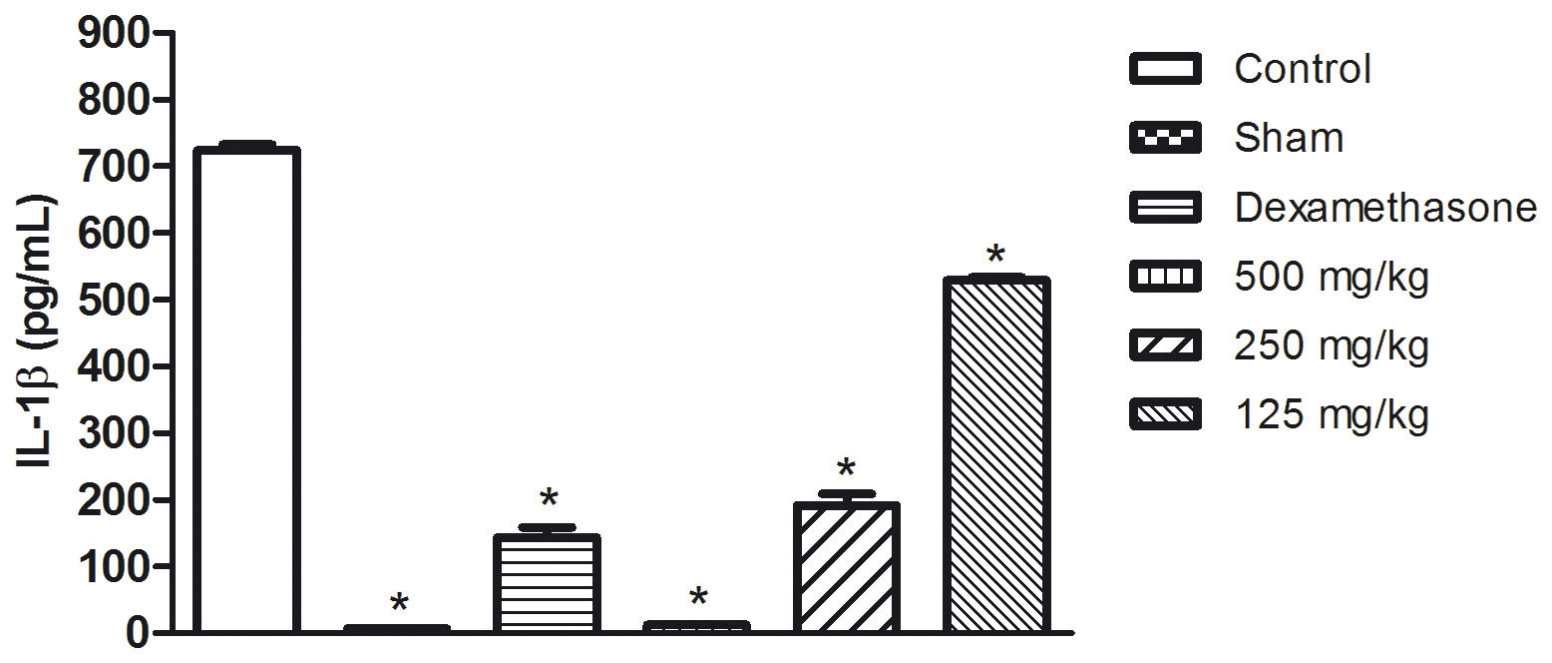
IL-1β (pg/mL) levels after the end of carrageenan-induced pleurisy assay in exudate of the pleural cavity of mice previously treated with aqueous seed extract. *p<0.05 in comparison with control by ANOVA followed by Newman-Keuls test.

**Figure 6:**
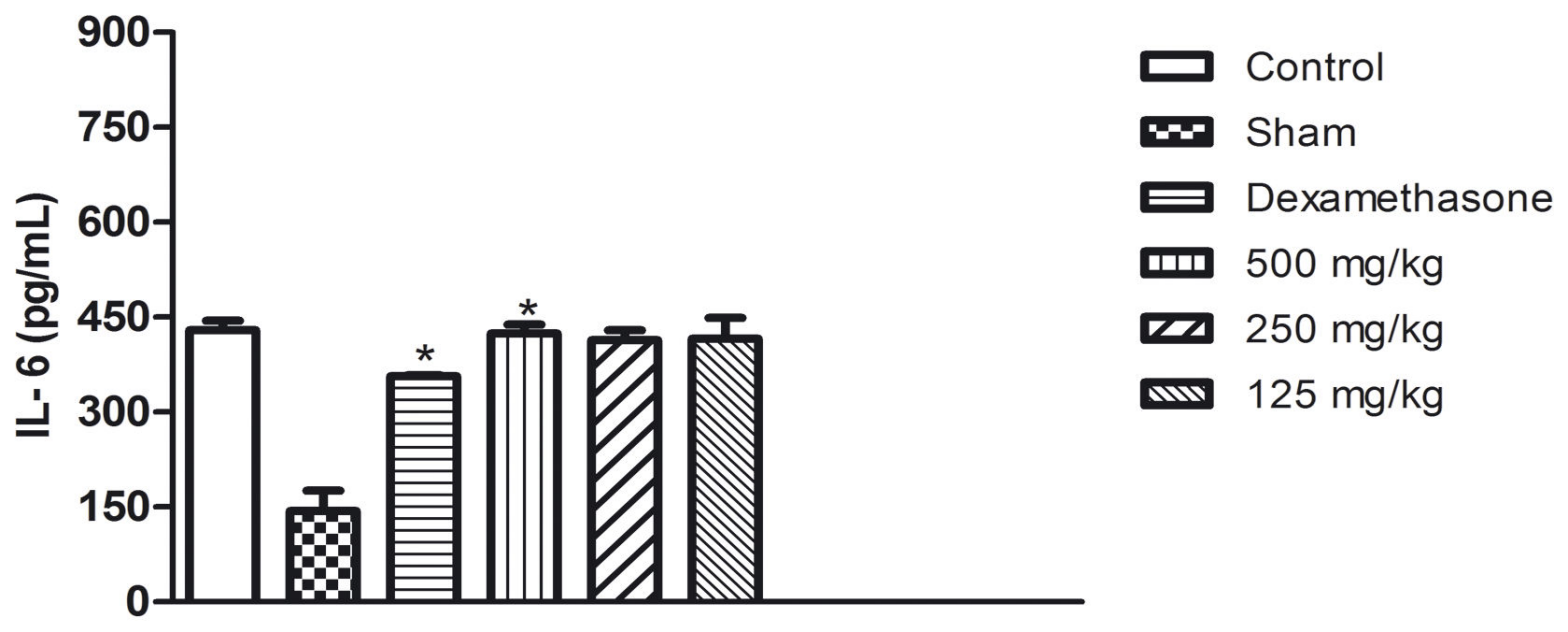
IL-6 (pg/mL) levels after the end of carrageenan-induced pleurisy assay in exudate of the pleural cavity of mice previously treated with aqueous seed extract. *p<0.05 in comparison with control by ANOVA followed by Newman-Keuls test.

The figures 4, 5 and 6 show the results of the measurement of the cytokine levels in the lung exudate from the *in vivo* anti-inflammatory assay. The aqueous seed extract also reduced the levels of TNF-α and IL-1β in a dose dependent manner, which is consistent with the effects observed on leukocyte migration. Despite the efficiency observed for doses of 250 and 500 mg/kg, the TNF-α levels in the animals treated with 125 mg/kg were not significantly different from the control group. IL-1β was slightly decreased in the animals treated with 125 mg/kg and was significantly reduced (p<0.05) in the animals that received the aqueous seed extract at doses of 250 and 500 mg/kg. The extract failed to reduce the levels of IL-6 at any of the doses tested. These results are in agreement with those from the *in vitro* assay. Dexamethasone, a corticosteroid, was the reference drug used as positive control in *in vivo* anti-inflammatory assay. As expected, dexamethasone was efficient in reducing leuckocite migration (Figure 2), NO production (Figure 3) and cytokine levels.

A clinical trial using *M. oleifera* seed powder in asthmatic patients showed improvement of the disease symptoms [66]. A subsequent mechanistic investigation using an alcoholic extract demonstrated that *M. oleifera* reduces mast cell degranulation, acts as a bronchodilator, possesses spasmolytic activity and reduces edema formation [5]. However, other studies designed to increase our understanding of the effects of the seeds of this plant on the inflammatory process, especially those using aqueous preparations, have not been conducted. 

The decrease in the levels of TNF-α induced by the aqueous seed extract appears to be required for the reduction of inflammation because this cytokine stimulates the production of other proinflammatory cytokines [67]. The reduced leukocyte migration observed may be related to the low levels of TNF-α and IL-1β because the first acts as a potent chemotactic agent, whereas the second stimulates the expression of adhesion molecules on endothelial cells [68,69].

**Figure 7:**
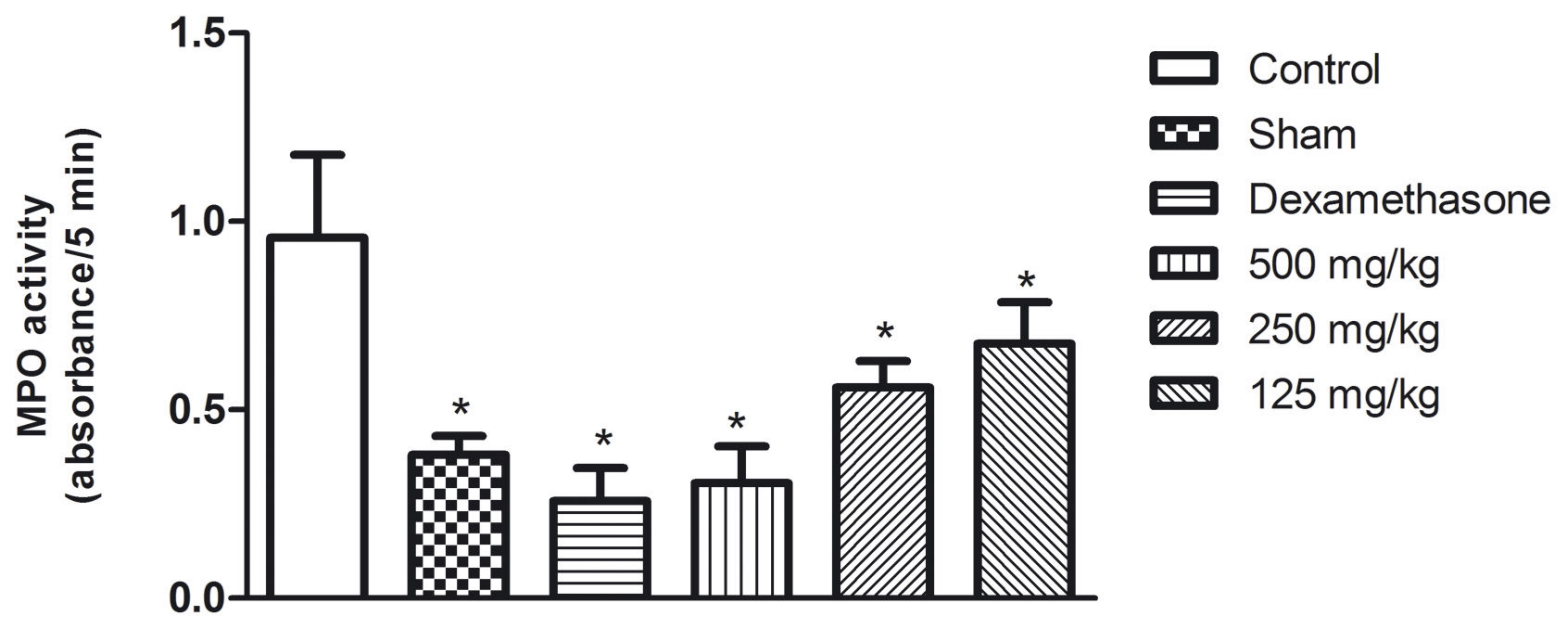
The effect of the aqueous seed extract on myeloperoxidase activity in the lungs of mice with carrageenan-induced pleurisy. The data are presented as the means ± standard deviation; n = 6 animals/group. *p<0.05 in comparison with the controls by ANOVA followed by Newman-Keuls test.

In accordance with the results obtained for leukocyte migration, the myeloperoxidase activity, an indication of the presence of neutrophils, was significantly reduced (p<0.05) in a dose-dependent manner in mice treated with the aqueous seed extract when compared with the controls (Figure 7). 

Myeloperoxidase, which is stored in granules of neutrophils, acts as an indirect indicator of the activity of these cells. Thus, the reduction in enzymatic activity, also in a dose-dependent manner, can be explained by the effect of the extract on cell migration. Consistent with this, we also observed reduced neutrophilic inflammation by histological analysis of the lungs of animals treated with the extract before the induction of inflammation in the pleural cavity.

**Figure 8:**
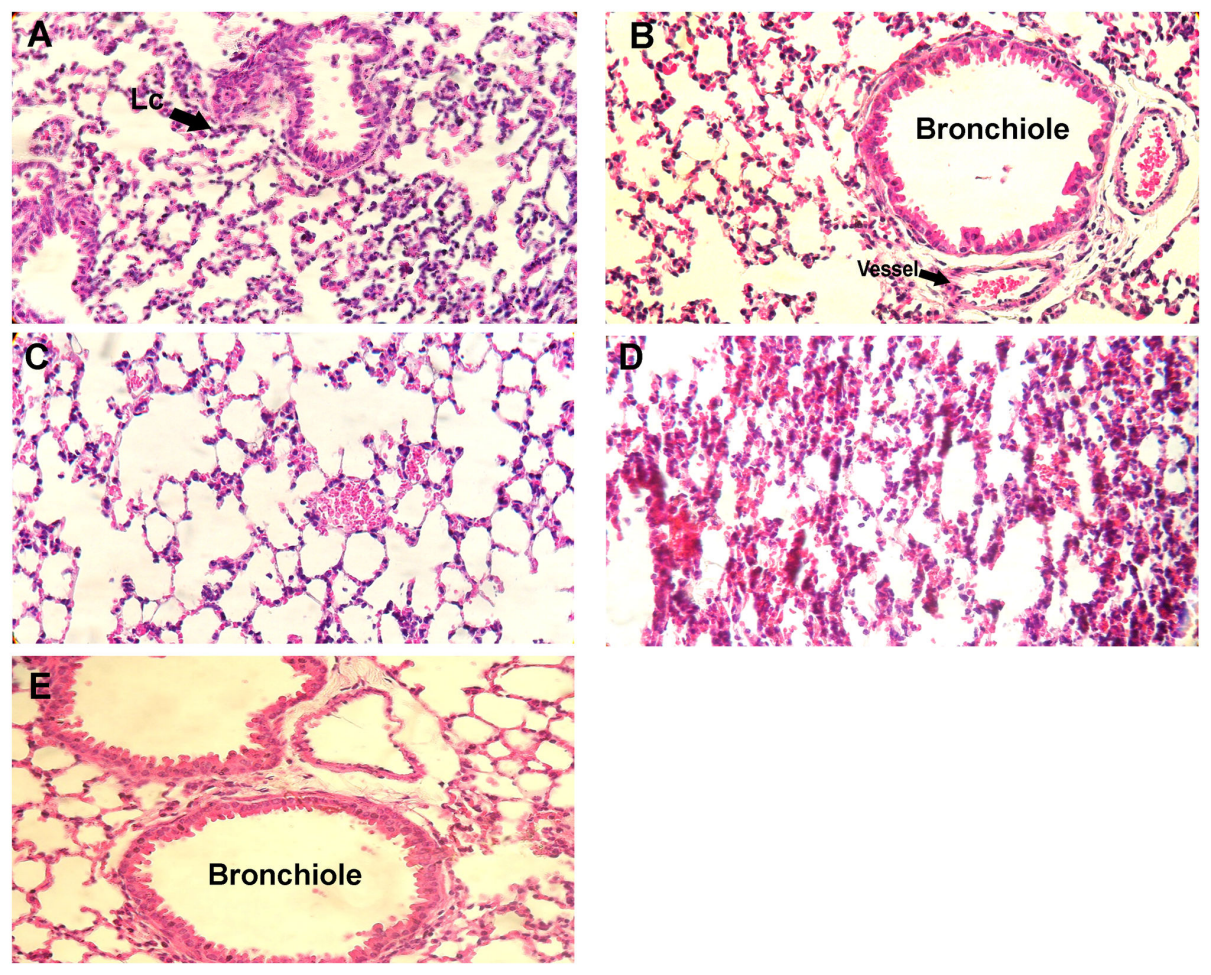
The effect of the aqueous seed extract on the infiltration of polymorphonuclear leukocytes into the lungs of mice with carrageenan-induced pleurisy. The histological sections were stained with hematoxylin-eosin (x 400). (A) Inflammation in the mice treated with carrageenan. (B) Reduction of the presence of PMN in the mice previously treated with the aqueous seed extract at 500 mg/kg. (C) Reduction of the presence of PMN in the mice that were previously treated with the aqueous seed extract at 250 mg/kg. (D) Extensive infiltration of PMN in the mice that were previously treated with the aqueous seed extract at 125 mg/Kg. (E) Normal mouse lung tissue. Lc = Leukocyte.

A histological analysis (Figure 8) of the lungs of animals with carrageenan induced pleurisy reveals the extensive infiltration of polymorphonuclear leukocytes (PMN), with a perialveolar distribution. The treatment with the aqueous seed extract at 500 mg/kg decreased PLMN infiltration and preserved the integrity of the alveoli and bronchi. The dose of 250 mg/kg also reduced the infiltration of polymorphonuclear cells when compared with the controls. Furthermore, the treatment with 125 mg/kg did not inhibit infiltration, corroborating the minimal reduction in the number of PLMN in the exudate from the pleural cavity. Thus, the reduction in the number of these cells was associated with a reduction in myeloperoxidase activity, NO levels and the production of TNF-α and IL-1 β, and the absence of interference in the production of IL-6. 

Plant lectins with anti-inflammatory activity have previously been described [23,70]. The aqueous seed extract contains lectin, as evidenced by the hemagglutinating activity and thus, the *in vivo* anti-inflammatory property of this extract may be mediated by lectins present in this preparation. 

## Conclusions

The aqueous seed extract and cMoL are potentially cytotoxic to peripheral blood mononuclear cells, while the diluted seed extract and WSMoL are not cytotoxic to these cells. None of the tested *M. oleifera* preparations promoted hemolysis of erythrocytes, and the aqueous seed extract did not cause systemic toxicity in mice. The seed extracts and lectins demonstrated *in vitro* anti-inflammatory activity on LPS-stimulated macrophages by regulating the production of cytokines and NO. The anti-inflammatory properties of the aqueous seed extract were confirmed using an *in vivo* model of acute inflammation; we observed a reduction in leukocyte migration, myeloperoxidas activity and the levels of TNF-α and IL-1β. Further studies using animal models should be performed to investigate the anti-inflammatory activity of diluted seed extract as well as isolated cMoL and WSMoL as well as provide a better understanding of their mechanisms of action.
